# HOXA10 is associated with temozolomide resistance through regulation of the homologous recombinant DNA repair pathway in glioblastoma cell lines

**DOI:** 10.18632/genesandcancer.16

**Published:** 2014-05

**Authors:** Jin Wook Kim, Ji Young Kim, Ja Eun Kim, Seung-Ki Kim, Hyun-Tai Chung, Chul-Kee Park

**Affiliations:** ^1^ Department of Neurosurgery, Seoul National University College of Medicine, Seoul National University Hospital, Seoul, Korea; ^2^ Division of Pediatric Neurosurgery, Pediatric Clinical Neuroscience Center, Seoul National University Children's Hospital, Seoul, Korea;

**Keywords:** HOXA10, temozolomide resistance, homologous recombination, EGR1, PTEN

## Abstract

Temozolomide resistance is associated with multiple DNA repair pathways. We investigated homeobox (HOX) genes for their role in temozolomide resistance, focusing on the homologous recombination (HR) pathway, and we tested their therapeutic implications in conjunction with O^6^-methylguanine DNA methyltransferase (MGMT) status. Two glioblastoma cell lines with different MGMT statuses were used to test the augmented anticancer effect of temozolomide with HOXA10 inhibition. In vitro experiments, including gene expression studies with RNA interference, were performed to verify the related pathway dynamics. HOXA10 inhibition reinforced temozolomide sensitivity independent of MGMT status and was related to the impaired double-strand DNA breakage repair process resulting from the downregulation of Rad51 paralogs. Early growth response 1 (EGR1) and phosphatase and tensin homolog (PTEN) were the regulatory mediators between HOXA10 and the HR pathway. Moreover, HOXA10 inhibition selectively affected the nuclear function of PTEN without interfering with its cytoplasmic function of suppressing the phosphoinositide 3-kinase/Akt pathway. The mechanism of HR pathway regulation by HOXA10 harbors another target mechanism for overcoming temozolomide resistance in glioblastoma patients.

## INTRODUCTION

Temozolomide has been a mainstay of chemotherapy for glioblastoma (GBM) for the past decade but still produces unsatisfactory clinical outcomes. Most GBM patients who are treated with standard therapy incorporating temozolomide eventually experience progression, and only 11% of patents remain progression free at 2 years [1]. The inescapable early treatment failure rate of standard treatment largely depends on temozolomide resistance. From the perspective of the drug mechanism of temozolomide, O^6^-methylguanine DNA methyltransferase (MGMT) has been proven to be associated with the prediction of the treatment effect [2]. However, there are further steps that lead the cancer cells to death after temozolomide treatment in relation to the DNA repair pathway, such as mismatch repair (MMR) and homologous recombination (HR) [3, 4]. Temozolomide cytotoxicity is initially mediated by the generation of O^6^-methylguanine from guanine, which can be repaired by MGMT [5]. However, unrepaired O^6^- methylguanine successively results in thymine mispairing during DNA replication, and these mispairs result in futile cycles of the repair process by the MMR system due to the persistence of O^6^-methylguanine in the template strand [5]. These futile cycles of the DNA repair process eventually cause double-strand DNA breaks, leading to cell apoptosis if the HR system functions inadequately [5]. Therefore, temozolomide resistance can be induced if MGMT is activated, MMR function is defective, or HR is normal. In addition to the established mechanism of temozolomide resistance by MGMT, reports have been published concerning inactivating mutations of MSH6 among MMR genes contributing to temozolomide resistance in GBM [6-8]. However, the incidence of MMR alterations in GBM is infrequent; thus, it is considered to be less important for temozolomide resistance [3, 9]. Otherwise, the contribution of HR to temozolomide resistance has been rarely studied, and little is known. We investigated temozolomide resistance and mechanisms to restore temozolomide sensitivity, focusing simultaneously on MGMT and HR.

To identify plausible targets for temozolomide resistance, we considered homeobox (HOX) genes, which showed a significant relationship between its dichotomized sub-classification with MGMT and survival in gene expression profiling studies, as previously reported [10]. HOX genes are a group of essential regulatory genes that normally control embryonic development and that should be in the silenced state in the adult central nervous system [11-13]. Recent studies have shown evidence of the aberrant expression of HOX genes in diverse cancers, including gliomas [14-18]. Although solid evidence exists concerning the role of HOX genes in oncogenesis and therapeutic resistance in gliomas, the exact mechanism remains unclear, and only a small number of recent studies have been published [9, 10, 18]. In the present study, we investigated the mechanism of HOXA10 regarding its role in temozolomide resistance using glioblastoma cell lines and tested the therapeutic implication of temozolomide resistance in conjunction with MGMT status. The result of the present study suggests a possible hypothesis for the temozolomide non-responders in MGMT-inactive GBM patients.

## RESULTS

### HOXA10 mediates temozolomide resistance independent of MGMT

**Figure 1:**
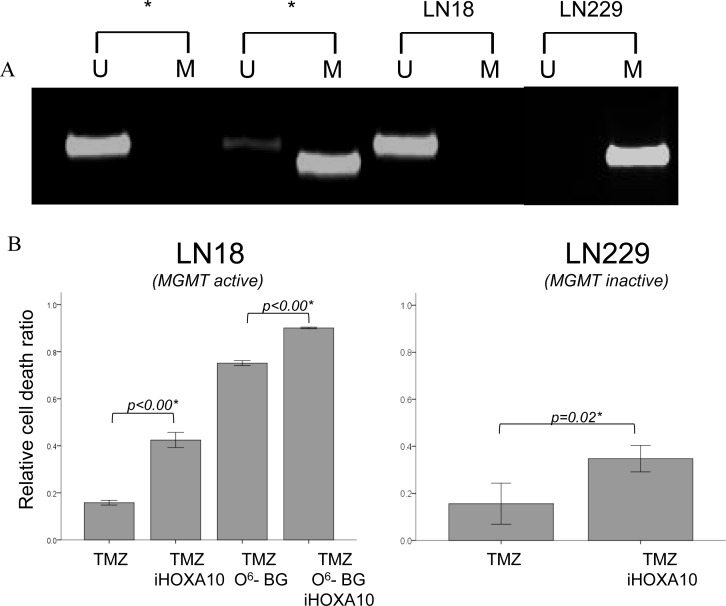
A. MGMT promoter methylation-specific polymerase chain reaction results. Each lane with an asterisk indicates control DNA for no methylation and methylation. The MGMT promoter is unmethylated in LN18 cells and methylated in LN229 cells. B. Cell viability results presented as the relative cell death ratio. Inhibition of HOXA10 shows an additive effect with temozolomide treatment in both cell lines, even after the inhibition of MGMT in LN18 cells.

The MGMT methylation status of the LN18 and LN229 glioma cell lines as measured by MSP revealed an unmethylated MGMT promoter (MGMT active) for LN18 cells and a methylated MGMT promoter (MGMT inactive) for LN229 cells (Figure 1A). Both cell lines displayed intact HOXA10 expression, which was successfully knocked down with iHOXA10. When the cell lines were treated with TMZ in combination with O^6^- BG and/or iHOXA10, significant differences in the cell death ratio were observed when iHOXA10 was added (Figure 1B). This additive effect of iHOXA10 on cell viability was independent of the MGMT status and created an added effect over MGMT inhibition.

### Screening for effectors of HOXA10

To identify HOXA10-regulated genes, we transduced LN18 cells with control siRNA or HOXA10-silencing siRNA (iHOXA10) and carried out microarray gene expression profiling. We compared the expression values of selected probe sets displaying average fold-changes of at least 2.0-fold, yielding 124 probe sets as being differentially expressed (15 up- and 109 down-regulated). Among them, only selected genes with RefSeq identifiers (NCBI Reference Sequence Database; http://www.ncbi.nlm.nih.gov/refseq/) are listed (Table 2). We then used the functional annotation tools within DAVID Bioinformatics Resources (http://david.abcc.ncifcrf.gov/home.jsp) to perform gene annotation enrichment [19]. This analysis indicated that early growth response protein 1 (EGR1) is a mediator for the functional category of transcription/cell division and chromosome partitioning among the listed genes. EGR1 showed a -3.09-fold change after iHOXA10 treatment. After thorough review of the functions of EGR1, we focused on previous reports indicating that EGR1 induces phosphatase and tensin homolog (PTEN) by regulating its promoter [20-22]. Moreover, evidence has shown that PTEN has novel nuclear functions, including transcriptional regulation of the Rad51 gene, whose product is essential for HR repair of DNA breaks [23, 24]. Therefore, we hypothesized that HOXA10 can regulate the HR system mediated by EGR1 and PTEN.

### HOXA10 selectively regulates the nuclear function of PTEN through EGR1

**Figure 2:**
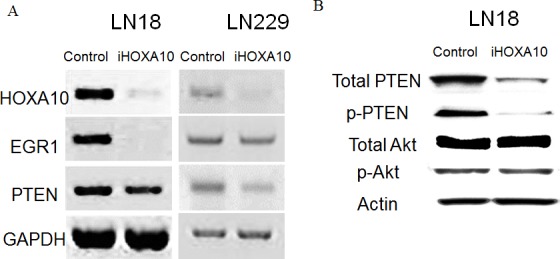

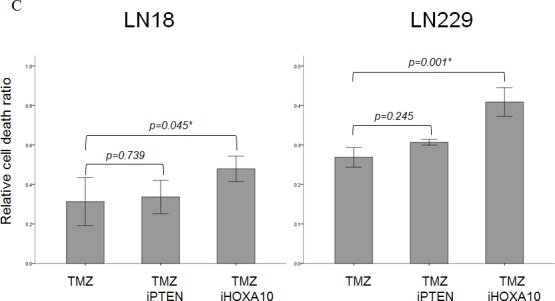
A. RT-PCR results after HOXA10 knockdown by siRNA. Reduced expression of EGR1 and PTEN is shown. B. Western blotting results after HOXA10 knockdown by siRNA. Despite the suppression of PTEN, the expression of both total Akt and phosphorylated Akt remained unchanged. C. Cell viability results presented as the relative cell death ratio. Direct inhibition of PTEN (inhibition of the cytoplasmic function of PTEN) had no influence on the anticancer effect of temozolomide, although inhibition of HOXA10 (indirect inhibition of the nuclear function of PTEN) had an additive anticancer effect with temozolomide in both cell lines.

After knockdown with iHOXA10, RT-PCR data showed significant suppression of EGR1 and PTEN in both LN18 (93% and 30% suppression) and LN229 (25% and 58% suppression) cells (Figure 2A). These results suggested that EGR1 and PTEN are the mediators regulated by HOXA10 status. However, suppression of PTEN induced by iHOXA10 did not affect the phosphoinositide 3-kinase (PI3K) pathway. The protein expression of total Akt and phosphorylated Akt showed no change, although PTEN was suppressed by iHOXA10 (Figure 2B). This finding implies that HOXA10 regulates only the nuclear function of PTEN without affecting its cytoplasmic function. This finding was also supported by the result of the cell viability test involving inhibition of PTEN directly with siRNA (iPTEN) that suppresses the cytoplasmic function of PTEN. No significant difference in cell death rate was noted whether iPTEN was added or not to TMZ, although significant differences were observed with the iHOXA10 and TMZ combination (Figure 2C).

**Table 1:** Primers used for PCR amplification

	Forward (5'→3')	Reverse (5'→3')	Amplicon length (bp)
HOXA10	AGGTGGACGCTGCGGCTAATCTCTA	GCCCCTTCCGAGAGCAGCAAAG	209
EGR1	CTGCACGCTTCTCAGTGTTCC	CGAGTGAGGAAAGGATCCGA	210
PTEN	TGGAAAGGGACGAACTGGTG	CACCTTTAGCTGGCAGACCA	289
Rad51b	TGTGGTGAAACACCCATCGT	TGTTCCACGAACACACAACCCA	255
Rad51c	TTTGGTGAGTTTCCCGCTGT	ACCCACCCTTAAAAGGAGAACA	399
Rad51d	GAATGGCGCTGATCTCTACGA	TCTCCTGGAAACCTGTTGGC	725
GAPDH	GCAGGGGGGAGCCAAAAGGG	TGCCAGCCCCAGCGTCAAAG	450

### Impairment of the HR system was observed with HOXA10 inhibition

**Figure 3:**
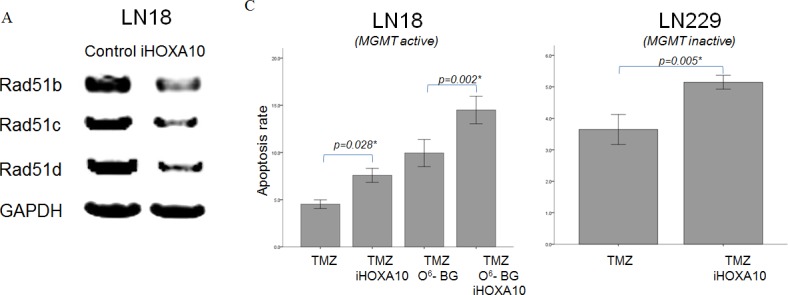

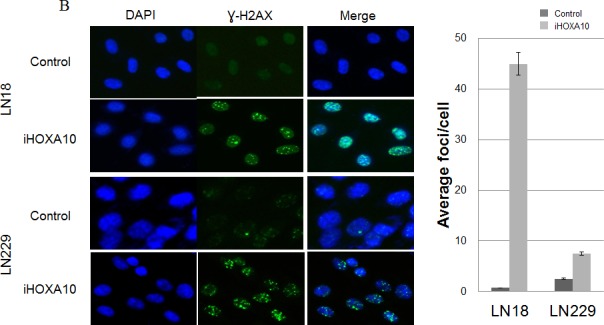
A. RT-PCR results after HOXA10 knockdown by siRNA. Reduced expression of Rad51b, Rad51c, and Rad51d is shown. B. Immunofluorescence image of γ-H2AX foci indicating double-strand DNA breakage. Increased numbers of γ-H2AX foci are observed with HOXA10 inhibition. C. Annexin V apoptosis assay shows a significant increase in apoptosis in the cancer cell lines by HOXA10 inhibition in combination with temozolomide.

We further validated the association of the regulatory activity of HOXA10 with the nuclear function of PTEN—namely, the maintenance of the HR system via the positive transcriptional regulation of Rad51 genes. Significant downregulation of all Rad51b, Rad51c, and Rad51d genes was found after HOXA10 knockdown (Figure 3A). Next, the formation of γ-H2AX at the site of double-strand DNA damage was checked. The kinetics of γ-H2AX foci is a well-accepted surrogate marker of the function of the HR system, which repairs DNA-double strand breakage [25]. A significant increase in the number of γ-H2AX foci was demonstrated in iHOXA10-treated cells (Figure 3B), indicating that inhibition of HOXA10 impairs the HR DNA repair system, potentially keeping cancer cells from escaping death after anticancer treatment. The apoptosis assay confirmed that a significantly increased number of cancer cells undergo apoptosis after treatment with both iHOXA10 and temozolomide (Figure 3C).

## DISCUSSION

Homeobox genes are essential developmental regulators, some of which are normally expressed during embryogenesis but are frequently up-regulated in cancer cells [26]. In the normal adult brain, most HOX genes are not expressed at all or are expressed at very low levels [13]. However, reports have demonstrated increased expression of HOX genes in brain tumors as well as other cancers from various organs [16, 26-32]. Murat et al. reported coordinated data of HOX genes and treatment resistance in GBM samples [10]. They found that high HOXA10 expression was predictive of resistance of treatment, including TMZ treatment, independent of the MGMT methylation status of the tumor [10]. The HOX genes aberrantly expressed in cancer cells are considered to be tumor modulators rather than tumor suppressor genes or oncogenes [26]. Recent studies concerning the functional analysis of HOX genes in glioblastoma also corroborate this assertion [9, 18]. Costa et al. demonstrated that HOXA9 plays oncogenic effects in GBM, such as inhibiting apoptosis and increasing cell proliferation, both of which can be reversed by inhibiting the PI3K pathway through an epigenetic mechanism involving histone H3K27 trimethylation [18]. They also showed that HOXA9 is an independent negative prognostic factor of survival; interestingly, HOXA9 expression remained a valid prognostic factor in the methylated MGMT promoter subgroup [18]. They suggested that the suppression of oncogenic HOXA expression by mTOR- or PI3K-targeted therapies can be a possible anticancer therapy for GBM patients [18]. Gaspar et al. reported a similar study with a more specific focus on treatment resistance [9]. They found high expression levels of the HOXA9/HOXA10 genes in pediatric GBM patient samples as well as a TMZ-resistant pediatric GBM cell line; high HOXA9/HOXA10 levels were related to shorter survival [9]. Temozolomide resistance in the high HOXA9/HOXA10-expressing GBM cell line was independent of MGMT status, and the PI3K pathway was considered to be an upstream regulator of HOX genes that can be targeted to overcome the resistance [9]. However, neither study investigated the downstream mechanism of temozolomide resistance induced by HOX genes.

**Table 2:** Identification of HOXA10-regulated genes in LN18 cells HOXA10-silencing siRNA versus control siRNA transduced cells are compared using Affymetrix GeneChip Human Gene 1.0ST Arrays. Probe sets with fold-changes of more than 2-fold are shown (3 up and 54 down probe sets).

	Gene Accession	Gene Symbol	log2 ratio (control vs iHOXA10)	Fold change
Up regulation				
1	NM_001771	CD22	1.37	2.58
2	NM_001042390	PTPN20A	1.38	2.60
3	NM_001143818	SERPINB2	1.51	2.85
Down regulation				
1	NM_001166292	PTCH2	−1.87	3.65
2	NR_002979	SNORA49	−1.79	3.46
3	NR_002960	SNORA20	−1.79	3.45
4	NR_003706	SNORA38B	−1.79	3.45
5	NR_000018	SNORD35A	−1.76	3.39
6	NR_003035	SNORA16A	−1.71	3.28
7	NM_014997	KLHDC10	−1.06	2.08
8	NM_015886	PI15	−1.69	3.23
9	NM_032290	ANKRD32	−1.65	3.15
10	NM_001964	EGR1	−1.63	3.09
11	NR_023343	RNU4ATAC	−1.60	3.04
12	NR_003137	RNU4-2	−1.57	2.97
13	NR_002963	SNORA24	−1.55	2.92
14	NM_001030	RPS27	−1.05	2.07
15	NR_002911	SNORA71A	−1.54	2.90
16	NM_001866	COX7B	−1.43	2.70
17	NM_005063	SCD	−1.43	2.70
18	NR_003041	SNORD13	−1.40	2.64
19	NR_003041	SNORD13	−1.39	2.63
20	NR_002749	SNORD45A	−1.37	2.58
21	NR_002962	SNORA23	−1.34	2.53
22	NR_002447	SNORD24	−1.30	2.47
23	NM_152997	C4orf7	−1.28	2.43
24	NR_000020	SNORD33	−1.26	2.40
25	NR_002748	SNORD45B	−1.26	2.40
26	NM_001030	RPS27	−1.19	2.28
27	NR_003018	SNORA71D	−1.18	2.27
28	NR_002580	SNORA3	−1.18	2.26
29	NM_004891	MRPL33	−1.17	2.25
30	NR_002569	SCARNA9	−1.04	2.06
31	NM_182511	CBLN2	−1.16	2.23
32	NR_004381	SNORD105	−1.14	2.21
33	NR_000021	SNORD32A	−1.14	2.21
34	NR_000019	SNORD34	−1.14	2.20
35	NR_003002	SCARNA13	−1.14	2.20
36	NM_019058	DDIT4	−1.12	2.17
37	NR_003017	SNORA71C	−1.12	2.17
38	NM_006111	ACAA2	−1.11	2.16
39	NR_000024	SNORD46	−1.11	2.15
40	NR_004380	SNORD104	−1.10	2.15
41	NM_000599	IGFBP5	−1.09	2.14
42	NR_002450	SNORD68	−1.09	2.13
43	NM_001030	RPS27	−1.09	2.12
44	NR_029707	MIR186	−1.08	2.12
45	NR_002922	SNORA13	−1.08	2.11
46	NR_000012	SNORA68	−1.07	2.10
47	NM_001170423	PRSS35	−1.07	2.10
48	NM_001030	RPS27	−1.07	2.10
49	NR_002961	SNORA22	−3.50	11.32
50	NR_002751	SNORD41	−2.93	7.63
51	NM_020299	AKR1B10	−2.41	5.31
52	NR_003925	RNU4-1	−2.02	4.05
53	NR_002753	RNU5F	−1.89	3.71
54	NM_016097	IER3IP1	−1.07	2.10

In the present study, based on our hypothesis and related experimentation, we confirmed the downstream mechanism of HOXA10 associated with temozolomide resistance. In summary, HOXA10 induces transcription of EGR1, which sequentially results in PTEN expression. PTEN in the nucleus then acts as a positive transcriptional regulator of Rad51 paralogs, which are essential for the maintenance of the HR DNA repair system, which can protect cancer cells from temozolomide-induced cytotoxicity. Thus, inhibition of HOXA10 can downregulate EGR1, PTEN, and Rad51 paralogs in serial order to interfere with the HR system of cancer cells, making the cancer cell more vulnerable to temozolomide treatment. These processes occur at the nuclear level, and thus, the inhibition of HOXA10 does not affect the tumor suppressor function of PTEN that occurs in the cytoplasm. Studies have proposed novel nuclear functions of PTEN, including transcription regulation, other than its classical role of repressing the PI3K/Akt pathway [23, 24, 33]. McEllin et al. have also shown that PTEN has a novel nuclear function of transcriptional regulation of the Rad51 gene [4]. They also mentioned that downregulation of HR due to PTEN loss would result in sensitivity to DNA alkylating agents or PARP inhibitors [4]. The role of EGR1 as a mediator of PTEN regulation has been proposed in multiple studies [20-22, 34]. The PTEN pathway is regulated at multiple different levels. Among them, p53, IGF2, PPARγ, and EGR1 are molecules that can directly act on the promoter of PTEN to activate transcription [35]. Taken together, all the above lines of evidence, including our results, support the integrity of the EGR1-PTEN-Rad51 axis for HR system regulation initiated by HOXA10.

TMZ has been reported to be a strong double-strand DNA break inducer with a potency more than 10-fold that of ionizing radiation [36]. Although studies have indicated that molecules comprising the HR system can be potential modulators of temozolomide cytotoxicity, the importance of the HR system for temozolomide resistance was not highlighted until recently [37-44]. Therefore, the status of HR system in cancer cells may likely be used a biomarker or target to determine the clinical response to TMZ treatment. Moreover, it is also important to consider the MGMT status together with the HR system status because the concepts of oncogenic addiction and synthetic lethality can be applied [45]. For example, the possibility exists for the augmentation of MMR and the HR system in cancer cells with MGMT in an inactive state, such as in GBM with a methylated MGMT promoter, which has potential to interfere with TMZ cytotoxicity. In that case, inhibition of the HR system itself may show an enhanced anticancer effect through sensitization to TMZ treatment. Our study indicates that the HOXA10 can be a good therapeutic target as well as a biomarker to overcome TMZ resistance in the management of GBM patients.

## MATERIALS AND METHODS

### Cell lines

The human glioma LN18 and LN229 cell lines were obtained and cultured in DMEM containing 10% fetal bovine serum and 5% antibiotics (streptomycin) in a humidified atmosphere of 5% CO_2_ and 95% air at 37°C. Both cell lines express wild-type phosphatase and tensin homolog (PTEN) as described previously [46]. We performed methylation-specific polymerase chain reaction (MSP) to confirm the methylation status of the MGMT promoter after DNA isolation and bisulfite treatment in these cell lines as described previously [47].

### mRNA expression

The primers used were designed using the primer-BLAST tool available on-line (http://www.ncbi.nlm.nih.gov/tools/primer-blast/). The primer sequence of HOXA10, EGR1, PTEN, Rad51b, Rad51c, Rad51d, and GAPDH are summarized (Table 1). Using these primers, reverse transcription-polymerase chain reaction (RT-PCR) was performed to evaluate their expression. Cell lines were lysed with TRIzol (Life technologies), and RNA isolation was performed using an RNeasy Mini Kit (QIAGEN, #74104). Total RNA was treated with DNase and then quantified by spectrophotometry. Additionally, cDNA was synthesized from 1 µg of total RNA using a reverse transcription kit (QIAGEN, #205311) according to the manufacturer's procedure. The RT-PCR reaction was carried out for 35 cycles, comprising 95°C for 5 min, 95°C for 30 sec, and 58°C for 30 sec with each primer set. RT-PCR products were resolved by 2% agarose gel electrophoresis, and the bands were quantified using image analyzing software (ImageJ v1.47; http://rsb.info.nih.gov/ij/).

### RNA interference

For small interfering RNA (siRNA) experiments, commercially available sequences targeting HOXA10 (iHOX; Sigma Aldrich, #SASI_Hs01_00172491) and PTEN (iPTEN; Dharmacon, #L-003023-00-0005) as well as nontargeting control siRNAs (Dharmacon, #D-001610-01-05) were used. At 70–80% confluence, the cells were transfected with siRNAs at the most efficient transfection condition determined by the NEON^®^ Transfection system (Life Technologies, #MPK5000). The cells were cultured in media without antibiotics to increase the siRNA transfection efficiency for 24 hours.

### Drug treatment and cell viability analysis

Normal and transfected cells were grown on 96-well plates at a density of 4×10^3^ cells per well for 24 hours. Temozolomide (TMZ; Enzo, #420-044-M100) and O^6^-benzylguanine (O^6^-BG; Sigma Aldrich, #B2292-50MG) were treated with a final concentration of 1000 µg/ml and 300 µg/ml for 24 hours, respectively. Cell viability analysis was performed using a Colorimetric Cell Counting kit-8 (CCK; Dojindo Molecular Technologies). Quantification of viable cells was performed by the reading of ultraviolet (UV) absorption spectra at 450 nm on a microplate 2 hours after adding 10 µl of CCK solution per well according to the manufacturer's instructions. All experiments were conducted in triplicate.

### Gene expression profiling

Samples of the LN18 cell line transduced with siHOXA10 as well as control siRNA for 24 hours were analyzed using Affymetrix GeneChip Human Gene 1.0ST Arrays (Affymetrix) to identify the gene expression changes before and after HOXA10 knockdown. Expression data were normalized using the robust multi-array average (RMA) method. Affymetrix Expression Console Version 1.1 (Affymetrix) was used to compare the group signals, and data were log-transformed (base 2) for parametric analysis. Differentially expressed genes were identified using significance analysis of microarrays (SAM) with the R package ‘samr’ (R 2.11.1).

### Protein detection

Whole protein extracts of the cells for western blotting were prepared using PRO-PREP lysis buffer (Intron, #17081), and protein concentrations were determined using the BCA protein assay (Thermo Fisher Scientific, #23227). Proteins were separated by 10% SDS-PAGE, blotted onto nitrocellulose membranes, and then probed with antibodies against total AKT (Genetex, #GTX121937, 1:3000 dilution), phosphorylated AKT (Genetex #GTX61708, 1:2000 dilution), total PTEN (Genetex, #GTX101025, 1:500 dilution), and phosphorylated PTEN (Genetex, #GTX61780, 1:1000 dilution). The membranes were then incubated with a goat anti-rabbit IgG secondary antibody (Jackson, #003318367, 1:4000 dilution) for 1 hour. The membranes were incubated in ECL-prime solution (GE Healthcare Amersham, #RPN2232) in the dark for 1 minute and then exposed under a fluorchemHD2 (Cell biosciences) for visualization.

### DNA double-strand break assay

The DNA double-strand break (DSB) rates were assessed by quantifying the rates of γ-H2AX foci. Approximately 5×10^4^ cells were seeded on coverslips after drug treatment and were cultured overnight in a 37°C incubator without antibiotics. The following day, cells were fixed with 100% cold methanol for 5 minutes and then permeabilized with 0.25% PBST for 20 minutes. Next, the cells were incubated in 5% skim milk to block non-specific protein-protein interactions and immunostained with a γ-H2AX primary antibody (Abcam, #ab22551) and an FITC-conjugated rabbit anti-mouse secondary antibody (Invitrogen, #A11029). The number of γ-H2AX foci with DAPI was determined using a fluorescence microscope (100× objective). Disrupted cells were excluded from the analysis. Foci counting was performed for an average of 50 cells. The mean number of foci per cell was scored, and the standard error of the mean was calculated.

### Apoptosis assay

For apoptosis assays, cells were harvested and suspended in annexin V-binding buffer (BD Biosciences, #556570) at a concentration of 1×10^5^ cells/100 µl. Next, 5 µl of annexin V-FITC and 5 µl of propidium iodide (PI) were added to each sample, and the samples were incubated in the dark at room temperature for 15 min. The samples were run through a FACScan flow cytometer (BD-FACSCalibur-2, #633488), and annexin V positive and PI negative cells were designated apoptotic.

### Statistical analysis

ANOVA and Student's t test were used to identify significant differences in the cell death rate, DSB assay, and apoptosis experiments. The results were analyzed using IBM SPSS Statistics software (version 19.0; SPSS, Inc.). Data are presented as the mean ± standard deviation (SD) of three or more separate experiments, and a P value of 0.05 was considered to be statistically significant.
